# Prenatal Maternal Lipopolysaccharide and Mild Newborn Hyperoxia Increase Intrapulmonary Airway but Not Vessel Reactivity in a Mouse Model

**DOI:** 10.3390/children8030195

**Published:** 2021-03-05

**Authors:** Margaret E. Kuper-Sassé, Peter M. MacFarlane, Catherine A. Mayer, Richard J. Martin, Y. S. Prakash, Christina M. Pabelick, Thomas M. Raffay

**Affiliations:** 1 Department of Pediatrics, Case Western Reserve University, UH Rainbow Babies & Children’s Hospital, Cleveland, OH 44106, USA; Margaret.Kuper-Sasse@uhhospitals.org (M.E.K.-S.); pmm71@case.edu (P.M.M.); caa4@case.edu (C.A.M.); richard.martin@uhhospitals.org (R.J.M.); 2 Department of Anesthesiology and Perioperative Medicine, Mayo Clinic, Rochester, MN 55905, USA; prakash.ys@mayo.edu (Y.S.P.); pabelick.christina@mayo.edu (C.M.P.); 3 Department of Physiology and Biomedical Engineering, Mayo Clinic, Rochester, MN 55905, USA

**Keywords:** airway hyperreactivity, vessel hyperreactivity, inflammation, hyperoxia, precision-cut lung slice

## Abstract

Maternal infection is a risk for preterm delivery. Preterm newborns often require supplemental oxygen to treat neonatal respiratory distress. Newborn hyperoxia exposure is associated with airway and vascular hyperreactivity, while the complications of maternal infection are variable. In a mouse model of prenatal maternal intraperitoneal lipopolysaccharide (LPS, embryonic day 18) with subsequent newborn hyperoxia (40% oxygen × 7 days) precision-cut living lung slices were used to measure intrapulmonary airway and vascular reactivity at 21 days of age. Hyperoxia increased airway reactivity to methacholine compared to room air controls. Prenatal maternal LPS did not alter airway reactivity in room air. Combined maternal LPS and hyperoxia exposures increased airway reactivity vs. controls, although maximal responses were diminished compared to hyperoxia alone. Vessel reactivity to serotonin did not significantly differ in hyperoxia or room air; however, prenatal maternal LPS appeared to attenuate vessel reactivity in room air. Following room air recovery, LPS with hyperoxia lungs displayed upregulated inflammatory and fibrosis genes compared to room air saline controls (TNFαR1, iNOS, and TGFβ). In this model, mild newborn hyperoxia increases airway but not vessel reactivity. Prenatal maternal LPS did not further increase hyperoxic airway reactivity. However, inflammatory genes remain upregulated weeks after recovery from maternal LPS and newborn hyperoxia exposures.

## 1. Introduction

Treatment with supplemental oxygen for neonatal respiratory distress is common following preterm birth [1]. Preterm infants are at elevated risk for developing later wheezing disorders [2,3,4]. While the mechanisms underlying airway hyperreactivity are multi-factorial [5], there is increasing awareness that modest exposures of the preterm lung to just several days of supplemental oxygen may contribute to short- and longer-term respiratory pathophysiology, even in infants without chronic lung disease [6,7,8]. Interestingly, newborn rodent models also show that exposure to mild and moderate neonatal hyperoxia (40–60% inspired O_2_) when compared to severe hyperoxia (>70% inspired O_2_) results in long-term increases in airway hyperreactivity [9,10,11]. Additionally, hyperoxia exposure is associated with pulmonary arterial hypertension in the preterm neonate [12,13]; and the effects of newborn hyperoxia in rodent models show variably increased pulmonary vessel hyperreactivity [14,15,16,17].

Antenatal inflammation and infection are associated with preterm delivery [18], with elevated risk for childhood wheezing in preterm infants [19,20,21]. Perinatal inflammation and infection is among multiple risk factors for pulmonary hypertension in premature neonates [12]. Lipopolysaccharide (LPS), or endotoxin, is the major component of the outer membrane of gram negative bacteria and produces an inflammatory response. Intraperitoneal LPS injection in pregnant rodents shows that while LPS does not enter the fetal compartment [22], there are measurable pro-inflammatory cytokines released in the pregnant female, the placenta, and variable detection in the amniotic fluid [22,23], with induced cytokine gene expression in the fetal pup [24]. Animal models have thus been used to investigate the interactions of perinatal inflammation and postnatal oxygen exposure with development of cardiopulmonary diseases [15,24,25,26,27].

The term mouse is in a saccular stage of lung development at birth [28]. Only postnatally does alveolarization begin with ongoing vessel and capillary angiogenesis; thus, the mouse has been used to model preterm human lung, airway, and vascular development [29,30]. Precision-cut living lung slice (PCLS) preparation allows for measurements of intrapulmonary airway and intrapulmonary vessel responses with intact parenchymal structures [31,32]. The objectives of the present study are: (1) to investigate the longer-term effects of fetal exposure to maternal inflammation (LPS) and postnatal mild hyperoxia during the saccular and early alveolar stages of lung development on intrapulmonary airway and vessel reactivity by PCLS preparations, and (2) to investigate changes in lung gene expression after room air recovery in this mouse model. We hypothesize that prenatal maternal LPS and postnatal hyperoxia exposure increase airway and vessel reactivity and that LPS and hyperoxia are associated with upregulation of inflammatory gene expression.

## 2. Materials and Methods

### 2.1. Ethics Statement

All procedures were carried out in accordance with the National Institute of Health (NIH) guidelines for care and use of laboratory animals and were approved by the Animal Care and Use Committee (assurance number: A-3145-01) at Case Western Reserve University, Cleveland, Ohio, USA: Protocol #20150066; Expiration 5/22/2021.

### 2.2. Mice and LPS Injections

Timed-pregnant C57BL/6 mice were purchased from a commercial vendor (Charles River, Willmington, MA). At gestational day 18 (E18), each pregnant female received a single intraperitoneal (i.p.) injection of either sterile saline or 0.1 mg/kg LPS (suspended in saline and briefly sonicated). Dams were then observed to give birth in our animal facility ~3 days later. Mice were maintained on standard 12 h light–dark cycles with ad libitum access to standard food and water.

### 2.3. Hyperoxia Exposure

**Figure 1:**
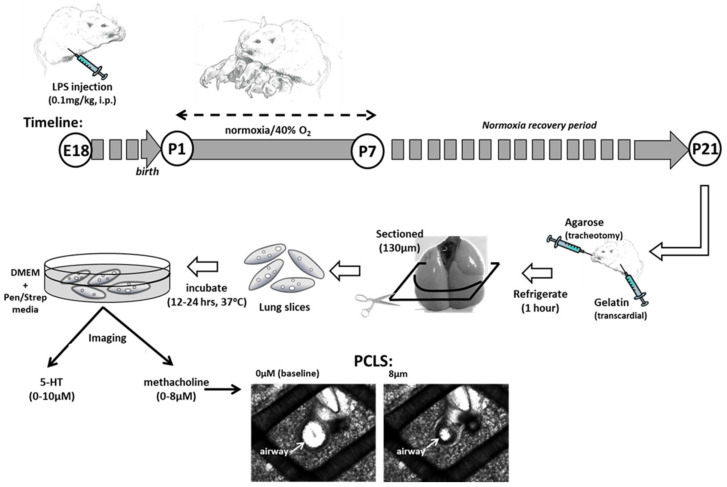
Mouse Model of Perinatal Inflammation and Hyperoxia Exposure. Pregnant dams received an i.p. injection of saline or lipopolysaccharide (LPS) on embryonic day 18 (E18). On postanatal day 1 (P1), pups were exposed to normoxia (21% O_2_) or mild hyperoxia (40% O_2_) for 7 days. All litters were returned to room air until 21 days of age. Precision-cut living lung slices (PCLS) were prepared and airway responses to methacholine challenge (shown) or vessel responses to serotonin challenge were recorded under video microscopy.

Immediately after birth (P0), the dams and their litters were randomly assigned into treatment groups to begin exposure to either continuous normoxia (21% O_2_) or hyperoxia (40% O_2_) the following day (P1). Mice were placed in a 38 L Plexiglas chamber designed with an incurrent and excurrent port to allow a continuous flow (4 L/min) of gas 24 h/day for 7 consecutive days as previously described [9]. Hyperoxia exposure was achieved by mixing air and 100% O_2_ using flow controllers to achieve the desired concentration of O_2_. Incurrent O_2_ levels were checked daily using an oxygen analyzer (MiniOX I; MSA Medical). At the end of the last day of exposure (P7), the nursing dam and pups were returned to room air for a further 2 weeks “recovery” period (Figure 1). At the end of the recovery period (P21), the mice were euthanized and prepared for measurements of airway and vessel hyperreactivity using the PCLS technique; additional mice were used for mRNA analysis on whole-lung homogenates.

### 2.4. Lung Slice Preparation and Reactivity Measurements

Two weeks after hyperoxia (or normoxia) exposures ended, P21 mice were sacrificed via anesthetic overdose [i.p. ketamine (100 mg/kg)/xylazine (10 mg/kg) cocktail] to prepare the lungs for in vitro measurements of airway reactivity to methacholine or vessel reactivity to serotonin. The mouse was placed supine for cannulation of the trachea. The cannula (PE tubing diameter 0.58 mm, Clay Adams, Sparks, MD, USA) was inserted through a small ventral incision of the neck and into the anterior most part of the trachea. The cannula was advanced ~3 mm and held securely in place with suture. Liquefied (40 °C) 2% agarose (Invitrogen, Carlsbad, CA, USA) in Hank’s Balance Salt Solution (HBSS) was gently injected to inflate the lungs (0.8 mL). To maintain vascular integrity, the chest was opened, the left atrium of the heart was cut and liquefied (37 °C) 6% gelatin in HBSS was gently injected into the right ventricle for transcardial perfusion of the lung vasculature until the gelatin draining from the cut left atrium was clear and free from blood. When the liquefied gel ran clear an ice cube was held over the left atrium to solidify the gelatin and block drainage. The mouse was then moved to 4 °C for 30–60 min to allow the agarose and gelatin to solidify. The entire lung was then removed, the lobe was embedded in agarose, placed on a vibratome (VT1000, Leica Microsystems, Wetzlar, Germany), and sliced into 130 μm sections. Slices were subsequently incubated in DMEM + Pen/Strep (Life Technologies, Carlsbad, CA, USA) media overnight (5% CO_2_; 37 °C) to melt the agarose and gelatin. The following day, the lung slices were rinsed in HBSS and mounted in an in vitro recording chamber for live imaging of responses to tissue bath perfused methacholine or serotonin [31].

For live imaging of individual airways or vessels, the slices were mounted in a well on a glass microscope slide, covered with a thin lightweight sheet of plastic mesh and a coverslip, held in place with silicone grease (Molykote, Dow Corning, Midland, MI, USA). The slide was mounted on a microscope (DMLFS, Leica Microsystems, Wetzlar, Germany) and perfused at 7 mL/min with solutions at room temperature using a perfusion pump (MPII, Harvard Apparatus, Holliston, MA, USA). Individual airways and vessels were identified under 5X magnification. A camera mounted on the microscope (Rolera Fast, QImaging, Surrey, BC, Canada) was used to capture images every 5 s. After an initial 3 min period of stabilization in HBSS, the chamber was perfused with increasing doses of methacholine or serotonin and video frames were recorded continuously. The extent of airway constriction in response to increasing doses of methacholine (0.25, 0.5, 1, 2, 4, and 8 µM) or vessel constriction in response to serotonin (0.1, 1, 10 µM) were determined at the end of a 2 min period of exposure at each dose by measuring changes in respective lumen area. Image analysis software (ImageJ software; NIH, Bethesda, MD, USA) was used to determine the luminal area (in pixels) at each dose of methacholine or serotonin to determine the extent of constriction. The greater the decrease in luminal area, the more reactive the airway or vessel. Data were normalized and expressed as a fraction of baseline lumen area as we have previously reported [9].

Individual airways and vessels were chosen at random and the responses to methacholine or serotonin were performed on one section at a time. Treatment groups consisted of 1–2 airways or vessels/animal consisting of 3–4 litters/group. Baseline lumen areas between 4000–7000 luminal pixels (1 μm = 0.39 pixels) were identified and chosen for analysis based on our prior work [33].

### 2.5. qRT-PCR

Whole lungs were harvested and snap frozen from different animals at 21 days of life. RNA was extracted from lung homogenates using RiboZolTM RNA Extraction Reagent (VWR Amresco, Radnor, PA, USA) and quantified by Nanodrop spectroscopy (Thermo Scientific, Waltham, MA, USA). cDNA was generated by reverse transcription using qScript cDNA synthesis kit (Quanta Biosciences, Gaithersburg, MD, USA). Quantitative real-time polymerase chain reaction (qRT-PCR) was performed on a StepOne PCR system (Applied Biosystems, Foster City, CA, USA) using commercial TaqMan gene probes (Life Technologies) for mouse IL-1β (Mm00434228_m1), IL-6 (Mm00446190_m1), iNOS (Mm00440485_m19), TGFβ (Mm01227699_m1), TNFα (Mm00443260_g1), or TNFαR1 (Mm00441883_g1) and house-keeping gene 18s (Mm03928990_g1) with PerfeCTa qPCR FastMix, UNG, ROX (Quanta Biosciences). Data are expressed as fold change to room air saline controls using the 2^(-ddCT) convention and StepOne software v2.3 (Applied Biosystems).

### 2.6. Materials

All chemicals and reagents were purchased from Sigma Aldrich, St Louis, MO and were of analytical grade unless otherwise noted.

### 2.7. Incusion and Exclusion Criteria

All live-born pups were randomized on P0 to room air or hyperoxia exposure and included in experiments. Baseline lumen areas measured as less than 4000 pixels or greater than 7000 pixels were excluded from analysis, as noted above. qRT-PCR was performed in replicate; any samples with a threshold CT value equal to or exceeding 36 cycles for the target gene and/or house-keeping gene were excluded from final qRT-PCR analyses.

### 2.8. Statistical Analysis

Changes in airway lumen area with methacholine or vessel lumen area with serotonin were analyzed using two-way repeated-measures analysis of variance (ANOVA) by doses and groups; changes in mRNA expression by qRT-PCR dCT were analyzed using one-way ANOVA by groups. Differences were considered significant at *p* < 0.05. Data are expressed as the means ± SEM.

## 3. Results

### 3.1. Airway Reactivity to Methacholine

**Figure 2:**
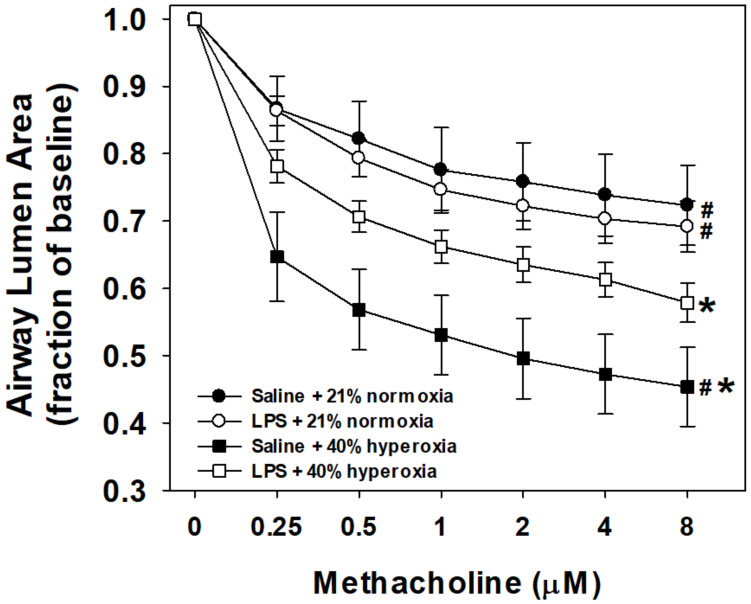
Airway responses to methacholine challenge in the in vitro living lung slice preparation from 21-day-old mice. PCLS airway responses to increasing doses of bath-applied methacholine in mouse pups that received one week of neonatal (P1–7) hyperoxia (squares) or normoxia (circles) and were born from pregnant dams that received an i.p. injection of saline (filled symbols) or LPS (open symbols) at E18 stage of pregnancy. Airways from hyperoxia-exposed mice pretreated with maternal saline were the most reactive (steeper slope, maximum airway contraction). Maternal LPS treatment and postnatal hyperoxia (open squares) significantly increased airway reactivity compared to control (saline + normoxia; filled circles), but attenuated the reactivity of hyperoxia alone (filled squares). Maternal LPS alone (open circles) did not differ in airway reactivity compared to control mice. Values are the means ± 1SEM. Significant differences in the dose response to methacholine: *vs. saline+normoxia (*p* < 0.05); #vs. LPS+hyperoxia (*p* < 0.05).

Airway reactivity to methacholine using the in vitro PCLS is shown by maternal treatment and postnatal exposure groups in Figure 2. Baseline airway lumen size did not significantly differ between groups. Airways from hyperoxia-exposed mouse pups pretreated with maternal saline (N = 9 pups) were more reactive than normoxia saline-treated mice (N = 9) as indicated by the notable decrease in lumen area in response to increasing doses of methacholine. Reactivity of airways from hyperoxia-exposed mice born to pregnant dams pretreated with LPS (N = 10) was significantly increased compared to normoxia saline-treated controls. However, reactivity was attenuated compared to the saline/hyperoxia-exposed mice. Airway reactivity to methacholine in normoxia-exposed mice was similar between mice that were from either maternal saline or LPS (N = 8) litters, suggesting that maternal LPS alone (without postnatal hyperoxia) did not impact airway reactivity compared to saline control mice.

### 3.2. Vessel Reactivity to Serotonin

**Figure 3:**
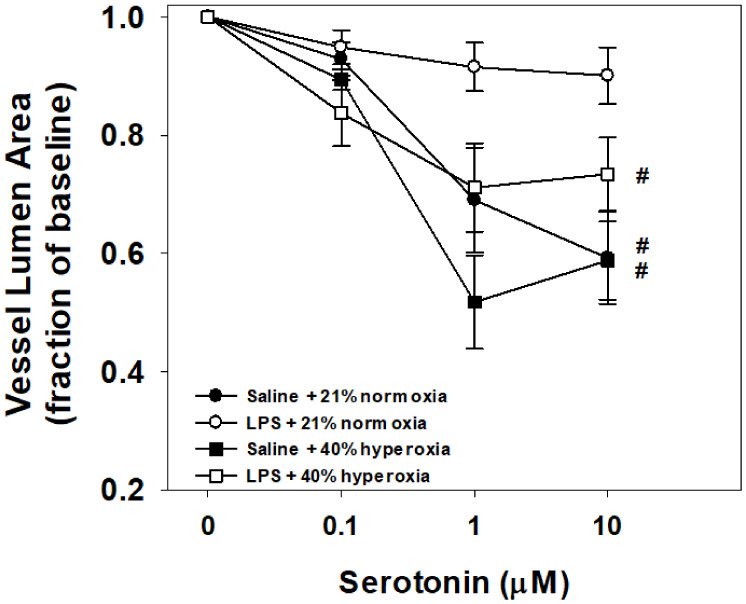
Vessel responses to serotonin challenge in the in vitro living lung slice preparation from 21-day-old mice. PCLS vessel responses to increasing doses of bath-applied serotonin in mouse pups that received one week of neonatal (P1–7) hyperoxia (squares) or normoxia (circles) and were born from pregnant dams that received an i.p. injection of saline (filled symbols) or LPS (open symbols) at E18 stage of pregnancy. Vessel reactivity of control (saline + normoxia; filled circles), hyperoxia alone (filled squares), and LPS with hyperoxia (open squares) did not significantly differ between groups. Prenatal maternal LPS alone (open circles) significantly attenuated vessel reactivity compared to all groups. Values are the means ± 1SEM. Significant differences in the dose response to serotonin: #vs. LPS+normoxia (*p* < 0.05).

Vessel reactivity to serotonin using the in vitro PCLS for each group is shown in Figure 3. Vessels from normoxia control mice pretreated with maternal saline (N = 8) were reactive to increasing doses of bath-applied serotonin. Similar degrees of reactivity were observed with the hyperoxia-exposed mice pretreated with maternal saline (N = 8) and maternal LPS (N = 8). Notably, vessels from pups born to pregnant dams that received LPS treatment without postnatal hyperoxia (N = 8) had attenuated reactivity compared to all other treatment groups, contracting 10% from baseline at the maximum serotonin dose of 10 μM. 

### 3.3. Lung mRNA Expression

**Figure 4:**
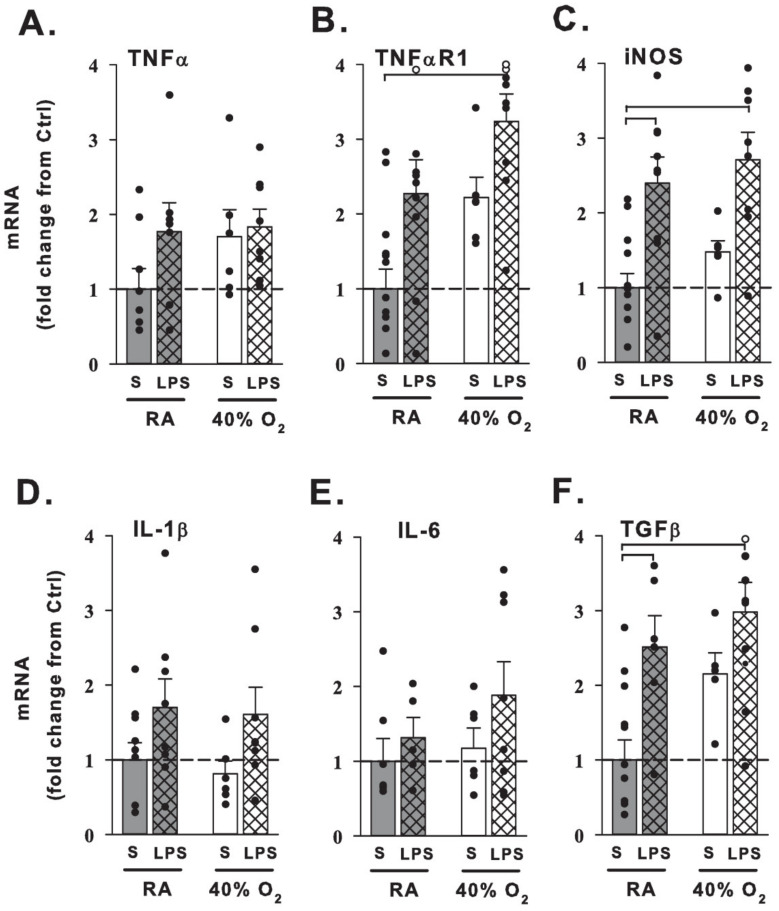
Changes in lung mRNA expression from 21-day-old mice. qRT-PCR of inflammatory-related molecules and receptors (**A**–**F**) from 21-day-old mice that received one week of neonatal (P1–7) hyperoxia (40% O_2_, white bars) or normoxia (RA, gray bars) and were born from pregnant dams that received an i.p. injection of saline (S, no cross-hatches) or LPS (cross-hatches) at E18 stage of pregnancy. Prenatal maternal LPS treatment with room air increased inducible nitric oxide synthase (iNOS; **C**) and transforming growth factor (TGFβ; **F**) mRNA expression in the lung. Hyperoxia mice pretreated with maternal LPS had increased tumor necrosis factor receptor (TNFαR1; **B**), iNOS (**C**) and TGFβ (**F**) mRNA expression compared to control mice. Data are expressed as fold change from saline + normoxia control mice (S+RA). Filled dots indicate individual animals and open dots exceed the 4-fold change of axis (TNFαR1: RA+LPS 5.0-fold change, 40% O_2_+LPS 4.2 and 4.2; TGFβ: 40% O_2_+LPS 4.7, respectively). Bar graph values are the means + 1SEM; brackets indicate *p* < 0.05 vs. control.

We next investigated the effects of prenatal maternal LPS and postnatal hyperoxia on lung mRNA expression at 21 days (N = 7–11 pups/group), shown as fold change in Figure 4. Mice raised in normoxia that were born to pregnant dams that received LPS exhibited increased inducible nitric oxide synthase (iNOS) and transforming growth factor β (TGFβ) mRNA expression compared to room air raised mice of dams prenatally treated with saline. Prenatal maternal LPS with newborn hyperoxia similarly increased iNOS and TGFβ, as well as elevated tumor necrosis factor α receptor-1 (TNFαR1) mRNA expression compared to controls. Neither LPS nor hyperoxia affected relative IL-1β, IL-6, or TNFα mRNA expression at 21 days of age.

## 4. Discussion

### 4.1. Maternal LPS and Hyperoxia Effects on Airway Reactivity to Methacholine

Airway reactivity to methacholine was significantly increased in PCLS after seven days of postnatal hyperoxia with room air recovery. These data replicate the previously observed increase in airway responses to methacholine after mild hyperoxia exposure, as reported by our group and others [9,10,11]. Prenatal maternal LPS treatment alone did not significantly change reactivity of the room air airways to methacholine. Contrary to our hypothesis; the effect of hyperoxia was not augmented by the perinatal exposure to inflammation. While still increased relative to room air controls, maternal LPS in the perinatal period attenuated the contractility of the hyperoxia-exposed airways to an intermediate extent, significantly less reactive than hyperoxia alone.

Other newborn mouse studies have demonstrated that the effects of combined exposures of perinatal LPS and postnatal hyperoxia variably affect baseline lung mechanics and also airway reactivity. Baseline in vivo airway resistance is increased and compliance decreased following both maternal i.p. LPS and postnatal hyperoxia when measured between 2 and 8 weeks of age [25,27,34]; potentially indicative of a stiffer or fibrotic airway. Royce et al. injected all C57BL/6 pregnant dams with i.p. LPS at a dose of 0.15 mg/kg at gestational day 14 (7 days instead of 3 days prior to birth) with postnatal exposure to 65% inhaled oxygen for 28 days (as compared to 40% for 7 days) [26]. Airway reactivity was measured using PCLS, which also showed increased reactivity to methacholine in the LPS and postnatal hyperoxia group compared to the room air and LPS group. However, PCLS experiments in hyperoxia alone were not reported. Faksh et al. measured in vivo methacholine responses on postnatal day 21 with a small-animal ventilator [25]. C57BL/6 pregnant dams were injected with i.p. LPS at a dose of 0.2 mg/kg (twice the dose used in this study) at gestational day 16 and exposed postnatally to 50% O_2_ for 7 days. Despite elevated baseline resistance, the combined exposures of LPS and moderate hyperoxia did not significantly differ at inhaled methacholine doses greater than 12.5 mg/mL when compared to saline room air controls. However, exposure to LPS alone showed maximal changes in measured resistance at baseline and at all methacholine doses. This change in baseline resistance corresponded with increased collagen smooth muscle deposition and *collagen-3* gene expression at 21 days. Finally, a temporal effect of prenatal exposure to inflammation has also been reported in other experimental animal studies sometimes showing progressive recovery from perinatal exposure to inflammation with or without hyperoxia [35,36,37,38]. Taken together, this supports our findings of a lack of additive synergy of the two exposures in regards to airway reactivity.

### 4.2. Maternal LPS and Hyperoxia Effects on Vessel Reactivity to Serotonin

Unlike the airways in this model, vessel reactivity to constriction with serotonin was not significantly increased after seven days of postnatal hyperoxia with room air recovery. Contrary to our hypothesis, this effect of hyperoxia was not augmented by perinatal exposure to inflammation via LPS injection to the pregnant dam. Interestingly, LPS exposure alone appeared to attenuate vessel reactivity to serotonin. When compared to our contrasting LPS airway physiology findings, these findings in vessels require further investigation.

Although hyperoxia is an acute vasodilator in the lungs [39], chronic exposure to oxygen is associated with increased pulmonary vascular resistance and pulmonary hypertension in preterm infants [12,13]. Balancing normoxia, hyperoxia, and hypoxia in the critically ill newborn is thus of high importance [40,41]. Infants with pulmonary hypertension and chronic lung disease show signs of pulmonary vascular disease during the first week of life and are sustained on supplemental oxygen for considerably longer durations beyond their initial course of neonatal respiratory distress syndrome [42]. While hyperoxia exposure of the preterm lung likely contributes to diminished vessel and capillary angiogenesis reported in human pulmonary hypertension [43], a proportion of infants have a component of elevated vessel reactivity [44,45]. As such, moderate and severe neonatal hyperoxia have been used to model rodent pulmonary hypertension with increased measures of pulmonary vessel reactivity [14,16,17]; to our knowledge the effects of mild hyperoxia (40% O_2_) on vessel responses have not been reported to date. Newborn mouse studies have also looked at the combined exposures of perinatal LPS and postnatal hyperoxia with variable results. Velten et al. reports perinatal LPS with hyperoxia results in cardiac dysfunction as early as 3 days of age in pups [24]. Conversely, Tang et al. reports that following perinatal inflammation, moderate 65% O_2_ (although not severe, >95% O_2_) in fact attenuated pulmonary hypertensive changes. However, vessel reactivity was not assessed [37]. Bui et al. reports that in vivo measurements of pulmonary vascular resistance were elevated with prenatal maternal i.p. LPS and postnatal hyperoxia (65% O_2_ × 28 days), although vessel reactivity assessed by PCLS did not differ between prenatal LPS and postnatal hyperoxia or room air [15]. Similarly, our mild hyperoxia exposure did not display elevated PCLS vessel reactivity with or without maternal LPS treatment. However, we did observe LPS alone resulted in significantly less responsiveness, a phenomenon that Bui et al. did not observe.

### 4.3. Maternal LPS and Hyperoxia Effects on Lung Gene Expression

Prenatal maternal LPS with or without hyperoxia upregulated lung iNOS and TGFβ gene expression at 21 days relative to saline room air controls. Hyperoxia with LPS resulted in elevation in TNFαR1 gene expression as well. Other cytokines genes measured (IL-1β, IL-6, TNFα) were not significantly increased with combinations of LPS and hyperoxia following room air recovery at 21 days.

Fetal exposures to inflammation as chorioamnionitis, maternal infection and fever with elevated acute phase proteins, tobacco use, or other pro-inflammatory factors coupled with immediate perinatal exposures to supplemental oxygen are thought to initiate injury to the lung epithelium and vascular endothelium [46,47,48]. As such, interventions targeting both inflammation and oxidative injury may be important in reducing prenatal lung disease [47,49,50]. Perinatal maternal inflammation has been shown to increase pro-inflammatory and fibrotic gene expression in the embryologic [24] and newborn [25] mouse lung. Furthermore, subsequent exposure to postnatal hyperoxia after perinatal LPS has been shown to increase multiple lung cytokines on day 3 of life [51] which begin to normalize by day 5 [25]. Inflammatory cell counts remain elevated following room air recovery [27,34]. In our model, profibrotic TGFβ and the inflammatory receptor TNFαR1 gene expression remained elevated following room air recovery, which may correspond to reports of persistent alveolar macrophage infiltration in other LPS/hyperoxia models [27,51] contributing to longer-term remodeling of the airways, vessels, and lung parenchyma. The potential effects of local nitric oxide from elevated iNOS gene expression on smooth muscle tone in a pathologic state [14,16,17] requires additional investigation in this model.

### 4.4. Limitations

These airway and vessel physiology studies demonstrate the complexities in the timing and severity of antenatal inflammation and hyperoxia exposure, suggesting a window in the perinatal period during which exposure to inflammation may indeed be beneficial to the hyperoxia-exposed airways rather than harmful. In a fetal lamb study, induced chorioamnionitis with endotoxin accelerates anti-oxidant activity [52], perhaps supporting a “priming” of defenses against mild hyperoxia which may be advantageous when combined [37]. Mechanisms through which LPS exposure would be potentially protective of hyperoxic airway reactivity are unknown and will require further investigation. As noted in the various double-hit models discussed, different exposures to LPS and hyperoxia are likely to show alternative effects. Additionally, while PCLS allowed for in vitro measurements of responses in individual airways and vessels with intact surrounding lung parenchyma, results of in vivo measurements may vary—particularly if there are baseline changes in lung mechanics [25,27,34] or pulmonary vascular resistance [15]. Finally, mRNA was extracted from whole-lung homogenates after a room air recovery period; single-cell sequencing or laser capture microscopy [25] at earlier time points may be more sensitive for detecting inflammatory gene changes in specific tissue compartments.

Our results suggest a pathologic effect of mild neonatal oxygen on airway reactivity that persists past infancy. We also show a novel effect of perinatal inflammation to attenuate airway reactivity. These effects were not observed in regards to the hyperoxia-exposed pulmonary vessels.

## 5. Conclusions

In this model, mild newborn hyperoxia increases airway but not vessel reactivity, while exposure to prenatal maternal LPS does not appear to add to this physiologic effect. However some inflammatory and fibrosis genes remain upregulated weeks after recovery in room air. We speculate that newborn exposures to even low supplemental oxygen for neonatal respiratory distress may increase the preterm infant’s risk for later wheezing disorders. It is unclear to what extent perinatal inflammation may contribute to augmenting or attenuating airway hyperreactivity. Further studies aimed at investigating the diverse underlying mechanisms of these physiologic findings are needed.

## Data Availability

The datasets used and/or analyzed during the current study are available from the corresponding author on reasonable request.
